# A proteogenomic update to Yersinia: enhancing genome annotation

**DOI:** 10.1186/1471-2164-11-460

**Published:** 2010-08-05

**Authors:** Samuel H Payne, Shih-Ting Huang, Rembert Pieper

**Affiliations:** 1 J Craig Venter Institute, 9704 Medical Center Drive, Rockville, MD 20850, USA

## Abstract

**Background:**

Modern biomedical research depends on a complete and accurate proteome. With the widespread adoption of new sequencing technologies, genome sequences are generated at a near exponential rate, diminishing the time and effort that can be invested in genome annotation. The resulting gene set contains numerous errors in even the most basic form of annotation: the primary structure of the proteins.

**Results:**

The application of experimental proteomics data to genome annotation, called proteogenomics, can quickly and efficiently discover misannotations, yielding a more accurate and complete genome annotation. We present a comprehensive proteogenomic analysis of the plague bacterium, *Yersinia pestis KIM*. We discover non-annotated genes, correct protein boundaries, remove spuriously annotated ORFs, and make major advances towards accurate identification of signal peptides. Finally, we apply our data to 21 other *Yersinia *genomes, correcting and enhancing their annotations.

**Conclusions:**

In total, 141 gene models were altered and have been updated in RefSeq and Genbank, which can be accessed seamlessly through any NCBI tool (e.g. blast) or downloaded directly. Along with the improved gene models we discover new, more accurate means of identifying signal peptides in proteomics data.

## Background

*Yersinia pestis*, a Gram-negative bacterium, is the causative agent of the bubonic and pneumonic plague. The pathogenic lifestyle of this microbe involves two distinct life stages, one in the flea vector, the other in mammalian hosts, primarily rodents [1]. *Y. pestis *recently speciated from *Y. pseudotuberculosis*, acquiring two pathogenic plasmids and a chromosomal pathogenicity island. Seven *Y. pestis *genomes have been sequenced to completion, along with five other *Yersinia *sequences. Numerous other *Yersinia *have been sequenced to draft quality.

Genome annotation is often divided into two sequential phases, finding genes and assigning function. Most prokaryotic genome annotation pipelines consist of automated gene finding, corroborated by limited homology comparisons. As such they lack any experimental validation of primary structure. Fundamentally, an accurate primary structure implies finding the correct start/stop of the gene, which may be erroneously predicted for 20% of genes in some bacterial and archaeal genomes [2,3]. But it also includes recognizing any true frame-shifting events, which must be delineated from sequencing errors or recent degeneration into a pseudogene.

A second benchmark for accurate primary structure is determining the mature protein sequence. Protein cleavage events (e.g. N-terminal export signal peptides, C-terminal LPXTG cell wall anchorage motifs) are particularly valuable clues for protein localization in the prokaryotic cell. Similarly, identifying differences between a mature virulence-associated protein and the nascent pre-protein can add valuable information as to how such a protein assumes its biological role in pathogenesis. Furthermore, modifications to amino acids (e.g., phosphorylation) implicate a protein in distinct and often transient biological processes (e.g. regulation of gene expression). None of these mature protein events are observable in DNA sequencing. They must be observed on the protein level.

A genome's annotation should be a dynamic working hypothesis, improved over time as understanding and knowledge increase [4,5]. Peptides observed from MS/MS experiments are an orthogonal data type from the DNA-centric evidences commonly used to predict protein-coding sequences (e.g. sequence homology, EST mapping, nucleotide and codon frequency metrics, etc.). Protein prediction using these extrinsic evidences, called proteogenomics, yields a more complete and accurate protein-coding catalog [6,7]. Specifically, proteogenomics can determine reading frame, translational start and stop sites, and the validity of short ORFs. In a variety of organisms, new insights from proteogenomics have consistently improved genome annotation [8-12].

In this work, we present a comprehensive proteogenomic analysis *Yersinia pestis KIM*. We discover non-annotated genes, correct the protein coding sequence of several of genes, remove many spuriously annotated ORFs, and make major advances towards accurate identification of signal peptides. Corrections have been updated in the RefSeq annotation of the genome (NC_004088). Through NCBI's peptidome, we have linked our experimental data directly to the genome annotation. Finally, we translate these improvements across other *Yersinia *genomes, to update annotation for the two other human pathogenic species.

## Results and Discussion

### Correcting Annotation Errors

**Figure 1:**
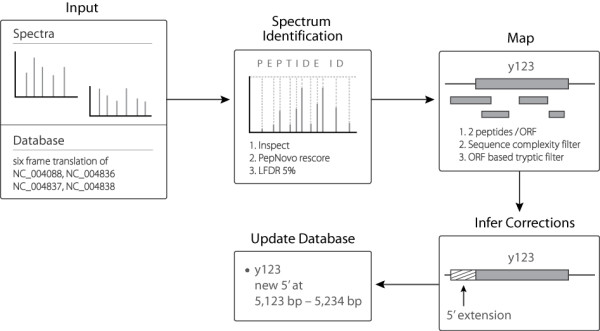
**Proteogenomics Pipeline**. MS/MS spectra and a protein sequence database are input for spectral identification by the Inspect program, which produces peptide/spectrum matches. PSM from Inspect are rescored with PepNovo, and filtered to an approximate 5% pvalue. Peptides are mapped onto the genome, with an additional layer of ORF-level filtering. Finally, peptides are compared to existing annotation. If peptide evidence shows an erroneous protein annotation, the correction is submitted to NCBI.

Following the data path outlined in the Methods (Figure 1), ~15 million MS/MS spectra from *Yersinia pestis KIM *were searched by Inspect and PepNovo against the six-frame translation of the genome. Confident peptide/spectrum matches were mapped onto the genome sequence, and used to infer annotation improvements. In total, we report 30,994 peptides mapping to 1302 proteins when requiring 2 peptides per protein. 1277 proteins (31% of the proteome) contain at least 1 uniquely mapping peptide. Of the 25 proteins lacking a unique peptide, the vast majority are an active transposase (IS285). The finding of an active transposon is intriguing given than transposons have been proposed as a driving force of *Y. pestis *genome evolution since its recent divergence from *Y. pseudotuberculosis *[13].

By mapping the proteomic data onto the genome, we are able to objectively determine the quality of the genome annotation. When peptides map outside of predicted proteins, there are two categories of annotation improvement. If the open reading frame lacks a predicted protein, the observed peptides are evidence for a novel gene. If the ORF contains a predicted protein and peptides map upstream, they are evidence for a 5' extension and new start site. Both of these situations necessitate an update to the genome annotation. Working with the RefSeq curators at NCBI, all of the instances discussed below have been updated, and can be accessed seamlessly through any NCBI tool (e.g. blast) or downloaded directly.

**Figure 2:**
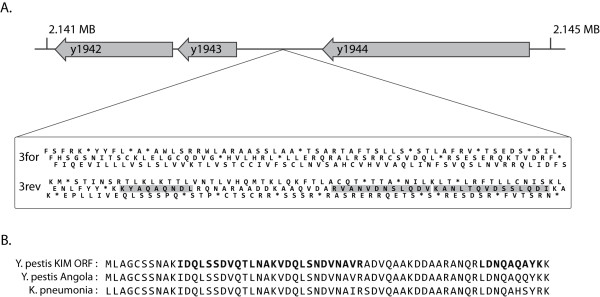
**Finding Unannotated Proteins**. (A) 700 bp genomic region between *Y. pestis KIM *proteins y1943 and y1944 lacked any annotation. Three proteomically identified peptides (boxed sequences) map to this region on the reverse strand. (B) Alignment of the open reading frame with homology to two other major outer membrane lipoprotein sequences. Peptides from proteomics data are bolded.

We find four ORFs which lack protein annotation, but have at least two uniquely mapping peptides (Figure 2). Three are easily recognizable proteins: major outer membrane lipoprotein between y1943 and y1944, two cold-shock proteins between y1817 and y1818 and y2562 and y2563. We also report an apparent *Yersinia *specific protein between y2035 and y2036. This protein, now named y5001, lacks homology to any currently described protein domain, and has no significant blast hits outside of the *Yersinia *genus. Finally, y3734 is predicted to be a pseudogene, an ABC transporter disrupted by insertion elements, indels, and nonsense mutations (Additional File 1, Figure S1). These mutations destroy several functionally relevant motifs, and almost certainly preclude proper biochemical function. However, we find two peptides mapping to the n-terminus of the region, providing evidence for its translation and presence in the cell.

**Figure 3:**
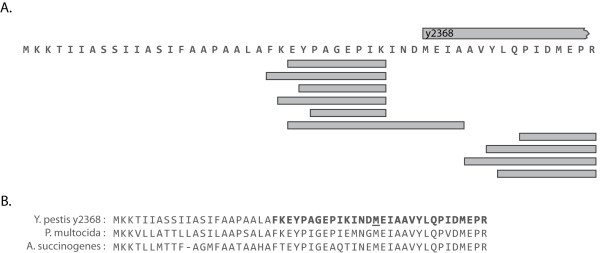
**Correcting Start Site Assignment**. *Y. pestis KIM *putative membrane protein y2368. (A) 6 peptides map upstream of the currently predicted start site. Sequence upstream of the peptides includes elements of a classical signal peptide: early basic residues, hydrophobic patch, and the 3 residue motif immediately before cleavage (see ref 17) (B) Alignment of the upstream region showing sequence conservation, peptides bolded.

The second major class of proteogenomic correction is start site annotation. Yersiniabactin thioesterase is an enzyme participating in the biosynthesis of a siderophore important for iron acquisition from the host. In our proteomics data we observed several peptides upstream of the current start for this gene, which required a 44 residue extension. Similarly, numerous peptides found upstream of the *Y. pestis *specific protein y0291 pointed to a 40 amino acid extension. Its lack of homology to any known domain and narrow taxonomic distribution demonstrate the utility of proteomic involvement in gene prediction. A third gene, y2368, highlights a subtle ramification of erroneous start sites, which is that functional elements within the n-terminus are obscured. y2368 is annotated simply as a 'periplasmic protein' with a CDD iron transport domain (cl01377). As expected we found this protein to be highly enriched in periplasmic as compared to cytoplasmic fractions [14], yet the n-terminus lacked localization motifs, e.g. a signal peptide. Six peptides mapped upstream of the annotated start site (Figure 3). Manual inspection of the region immediately upstream from these peptides found a strong signal peptide sequence motif.

Two proteins with erroneous start sites are special cases and widely mispredicted in bacterial genomes. Peptide chain release factor II, prfB, often contains a ribosomal +1 frame shift. As with the KIM genome, erroneous annotations of this protein typically contain only the c-terminal ORF but exclude the true n-terminus (Additional File 1, Figure S2). Our proteogenomics pipeline recognized peptides in both ORFs and the two were stitched together. We also found peptides upstream of infC, protein chain initiation factor IF-3, which utilizes an ultra-rare start codon ATT [15].

### Signal Peptides

**Figure 4:**
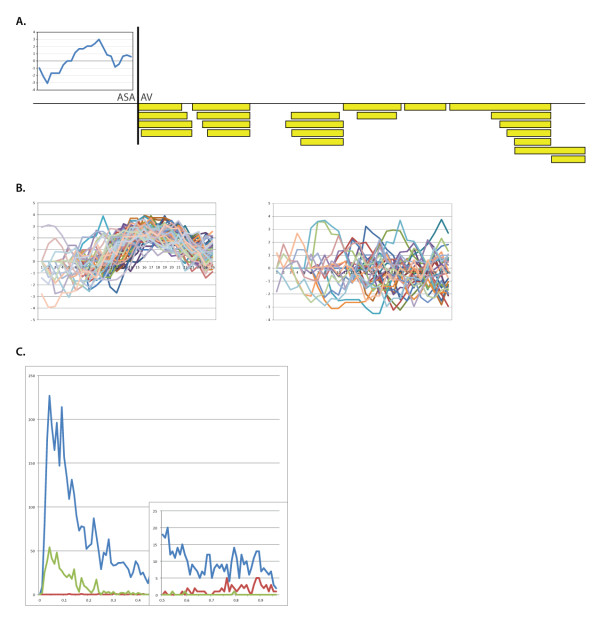
**Signal Peptide Identification**. (A) *Y. pestis KIM *znuA, a periplasmic zinc transporter is shown with the key elements of a signal peptide. The left graph inset is a hydropathy plot showing a stretch of hydrophobic residues (positive values). The cleavage is marked by '|' between ASA (the signal peptidase recognition motif) and AV (the beginning of the mature protein). Peptides observed in our dataset are marked with boxes. (B) Hydropathy plots from putative signal peptides with a hydrophobic patch, and without. Note that for signal peptides with a patch (left) there is a clear preference for the location of the motif - see discussion in the text. (C) Comparison of signalp scores with proteomic observation. The score of all proteins in plotted in blue. The score of proteins confirmed as containing a signal peptide is in red. The score of proteins rejected by proteomics is in green.

Proteins exported from the cytoplasm through the Sec-dependant pathway contain a short sequence essential to targeting and export (Figure 4). The ~20 residue motif, or *signal peptide*, is located at the n-terminus of the full-length protein. The signal peptide helps target the protein to the membrane, where it is temporarily anchored by a patch of hydrophobic residues. A three amino acid motif following the hydrophobic patch is recognized by the signal peptidase enzyme; the protein is cleaved and the c-terminal portion of the protein is released into the periplasm. The signal peptide, still anchored in the membrane is rapidly degraded by the signal peptide peptidase. The Sec-dependent pathway is separate from other export pathways, such as the Type III secretion system. Signal peptides are also present in proteins exported through the Twin Arginine receptor-mediated pathway, but are typically longer and include an additional motif prior to the hydrophobic patch.

Proteolytic cleavage of proteins *in vivo *can be recognized by proteomics by their atypical peptide endpoints. Spectra used in this report were generated from proteins digested with trypsin. Thus, we expect most identified peptides to be fully tryptic. Previously Gupta and colleagues postulated that signal peptides could be discovered simply by identifying non-tryptic peptides [16]. In their analysis, if the first observed peptide in a protein had a non-tryptic n-terminus and was within 17-55 residues of the start site, then it was a considered evidence of signal peptide cleavage. 202 Yersinia proteins fulfill these two requirements. We extend Gupta's criteria to include critical biological motifs within the signal peptide: the hydrophobic patch and cleavage motif [17]. Filtering out proteins lacking these new requirements, we report 82 proteins with observed signal peptide cleavage (Additional File 2, Table S1). These proteins also contain other common signal peptide features: prevalence of LL doublets and early basic residues. Furthermore, we noticed that the hydrophobic patch had a similar placement within the signal peptide for all validated proteins (Figure 4B). This location is consistent with the patch's structural purpose, i.e. membrane anchoring and exposure of the cleavage motif at an appropriate distance from the membrane. Finally, we report that many of the sequences contain not simply early basic residues but contain Met-Lys as the first two residues in the protein sequence. 34 of the 82 proteins start in this manner. An additional 15 have Met-Lys internally which could be the true start, if this trend is seen as a general pattern.

Twenty proteins contain signal peptides longer than 30 residues, which is atypical. When we compared these sequences to close homologs within the gamma-proteobacteria, 13 of them could be better predicted at an alternative start site downstream of the current annotation. This shorter version of the protein would not only have better agreement with homologous genes, but also the characteristics of signal peptides noted above (not just length). An additional four long signal peptides appear to contain the twin arginine motif, which are characteristically longer than those exported through the Sec-dependent pathway.

The set of secreted proteins within a genome is often computationally predicted. To compare our proteomic observations to such predictions, we ran signalp on all proteins in the genome. We plot the score for proteomically validated signal peptide containing proteins against the background of signalp's score distribution (Figure 4C). We also plot proteomically rejected signal peptide containing proteins (see Methods). The proteomically observed and rejected proteins separate very clearly, with the positive set scoring well above the suggested cutoff. Furthermore, proteomics and signalp generally agree on the exact residue of cleavage.

### Dubious Genes

**Figure 5:**
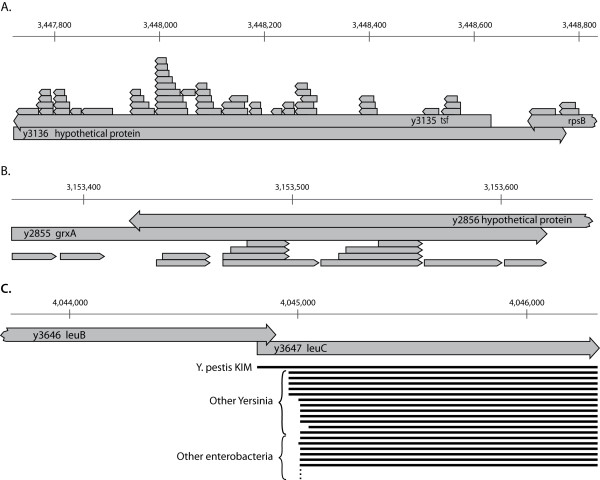
**Overlapping Genes**. (A) At 3.44 M on the KIM chromosome, a locus showing a dubious gene (y3136) overlapping two real genes, both of which have strong proteomic evidence (peptides boxed above the gene, showing orientation). Note that y3135 is completely overlapped by the dubious gene. (B) At 3.15 M two predicted proteins overlap by 206 nucleotides. There is strong proteomics evidence for the existence of glutaredoxin 1 (14 peptides), and no support for the hypothetical protein on the opposite strand. (C) At 4.04 M leuB and leuC have an overlap of 67 bp. An alignment of non-redundant leuC sequences from enterobacteria shows that the KIM version is predicted to be longer than any other close homolog.

In the *Yersinia pestis KIM *genome, there were over 200 genomic loci with a >50 bp overlap between two protein coding genes. Such a substantial overlap is unusual, especially considering that 10% of the proteome (~400 proteins) falls into this category. We viewed these *conflicted loci *as unlikely to be correctly annotated. For the 46 loci covered by proteomics, we manually reviewed the evidence supporting the existence of either gene. 38 loci contained a dubious gene (Figure 5). In over half of the instances, the loci contained genes with 100% sequence overlap. The equivocal nature of dubious genes was witnessed by narrow phylogenetic distribution, poor and seemingly random sequence conservation, and weak computational justification noted in the original genome submission (see Methods). The remaining 8 loci were in conflict due of an overly extended 5' on one or both of the genes (Figure 5C). Working with RefSeq curators at NCBI, the dubious genes have been removed. Analysis of the remaining ~150 conflicted loci is being addressed in future work.

### Improvement of Related Genomes

As a final step in our analysis, we used our proteomics data to improve the annotation of other *Yersinia *genomes. Published in 2002, KIM was the second annotated *Yersinia *and served as a source for subsequent genome projects [18]. Thus errors in KIM's annotation are likely to show up in other genomes. We created one-to-one orthology maps for proteins from 21 *Yersinia *genomes, and used our peptide mappings from the KIM dataset to evaluate gene models. Transferring proteogenomic improvements is complicated by both legitimate differences between strains/species, but also artifacts from sequencing and assembly. Our approach was to be conservative, and only change gene models where the evidence was clear.

We started with the removal of dubious genes, resulting in the deletion of 46 accessions. Remembering that each dubious gene was part of a conflicted locus, we checked to make sure that the true gene was present. At two loci we found that the true gene was missing, excluded from the original annotation by the dubious gene. We created new gene models for each of these. Three additional new genes were created, arising from the transitive annotation of the novel KIM genes. The two cold shock proteins were missing from *Y. pseudotuberculosis IP 31758*. The major outer membrane lipoprotein, lpp, was missing from *Y. pestis Pestoides F*.

To correct start sites in other *Yersinia *genomes, we applied the n-terminal most peptide from a gene in KIM to members of its ortholog set. If an ortholog was too short to include this peptide, we tested whether an extension could be made to accommodate. In 43 instances, this extension was trivial - clear homology up to and including the start codon. For 22, an extension could not be made due to indels or mutations shortening the reading frame. Finally, 10 were difficult cases where we could extend the reading frame to an upstream start codon, but homology was unclear. In these cases, we did not alter the genome annotation. This was particularly problematic in *Y. enterocolitica*, which is the most divergent genome used in our comparison.

## Conclusions

Each primary genome annotation is unique. From parameters and cutoffs, to pragma or data sources, the differences between genome annotation pipelines are non-trivial. When the number of places producing genome annotation is taken into consideration, the disparities become ever greater. When annotations disagree, there is rarely experimental proof as to which is right. Proteogenomics is a fast and effective way of improving genome annotation. By comparing experimental proteomics data with a genome annotation, we root ourselves in the observed proteome. In *Y. pestis KIM*, we show several instances of misannotation. As an isolated event, a few misannotations here or there may not seem overly significant. But as we have shown, misannotation is multiplied in future genomes.

Given the size of our data, we were somewhat surprised to find only four novel genes and five misannotated start sites. There are three potential explanations for this. First, gel-based proteomics is inherently limited in its sampling depth, as compared to the MudPIT experimental set up. This reason is not entirely satisfactory, as we report coverage for >30% of the proteome. Second, *Yersinia *is closely related to *E. coli *and thus benefits from its well curated genome. Finally, and most importantly, we note the tendency for overprediction within the annotation. This is evidenced by the extreme number of overlapping genes (many of which were proven to be dubious) and the frequent occurrence of genes which are longer in KIM than their homologs in other genomes. As proteogenomics relies on underprediction to find corrections (meaning that we identify a region of the genome which is not predicted to be coding but should be), this tendency diminishes our power for annotation improvement. Yet we are still able to significantly improve and enhance the annotation, especially by incorporating our data back to public repositories.

Misannotated start sites are regularly found in proteogenomic surveys. As reported by Salzberg [19], genomes annotated before 2006 had a ~ 20% error rate for start site assignment. The five cases discovered here highlight necessity of protein-based genome annotation, and the difficulties surrounding purely computational predictions. The most difficult situation to automate is the usage of exceptional or rare start codons. Our example here is IF-3 which utilizes the ATT codon, and has been found misannotated in other proteogenomics reports [16]. We note that this rather fundamental gene is mispredicted in most enterobacterial genomes. The full extent of rare start codon usage is not known, although a recent report proposed several new rare codons in Deinococcus [20]. A second category of difficult genes are those unique to a genus or species, often the more biologically interesting set of proteins. Without broad sequence distribution across taxa, comparative genomics is not effective. A final case is programmed ribosomal frame-shifts, such as *prfB*. In the *Yersinia *genus, *prfB *is annotated in two predominant forms, a full length protein, and one which lacks ~60 amino acids from the n-terminus (Additional File 1, Figure S2). The +1 frame shift required to maintain proper reading frame is simply not considered in most annotations.

Our analysis of the observed proteome reveals the difficulties surrounding the annotation of pseudogenes. Whether shortened by nonsense mutations or disrupted by indels, pseudogene annotation is inconsistent. In our orthology clusters, we observed genes split into multiple ORFs variously annotated as a truncated protein, pseudogene, unannotated, or a protein without comment. Second, we highlight the implication of marking a region as a pseudogene, which gives the impression that this genomic locus should be ignored. However both here and elsewhere [21] proteomics has revealed that many pseudogenes are 'alive' - translated and present in the cell. The truncated ABC transporter y3734 observed here almost certainly does not have the expected function. How should annotation transparently and concisely express the evidences and caveats for this phenomenon?

As we mapped peptides onto the genome, we noticed that some loci contained multiple gene predictions with substantial overlap. 80% of the time, this was caused by the prediction of a dubious gene, which we have removed from the annotation. Unfortunately, these dubious genes polluted public repositories, acting as a source for future annotations. As we sorted through the conflicted loci, we discovered two additional problems which hamper exclusively computational genome annotations. In 24 loci where one gene was completely overlapped by another (Figure 5A), six times the smaller gene was the true gene. This presents a problem for some gene calling pipelines, where the longest ORF in a region is selected to the exclusion of other overlapping gene possibilities, which in these cases includes the true gene. Second, we observed seven loci where the overlapping genes are both hypothetical. In such situations, proteomics can provide experimental validation to unambiguously determine the correct gene model.

Aside from highlighting annotation errors, proteogenomics can supplement annotation by providing value-added information about the mature functional protein. In our effort to reliably observe proteolytic cleavage events in proteomic data, we have introduced the new requirements for validating signal peptide cleavage. By filtering out proteins lacking a hydrophobic patch and the signal peptidase cleavage motif, we remove many spurious assertions. Unlike previous reports, we do not find a great discordance in the proteins identified by proteomics and computational predictions. Studies in *Shewanella *found 28% of proteins observed by proteomics are missed by signalp, and 26% of computational predictions are refuted by proteomics [16]. Our results are much more tempered, with 8% false-negatives and < 1% false-positives. There are two likely causes for the differences in our findings: the phylogenetic differences between *Yersinia *and *Shewanella *and new filters introduced in this work. The large excess of false-positives in *Shewanella *(i.e. proteins predicted by signalp but refuted by proteomics) may be attributable to the training set of proteins used for signalp, which is heavily overrepresented by enterobacteria (e.g. *E. coli*). *Yersinia*, another enterobacterium, is very closely related to *E. coli *and is more similar to data in the training set. The excess of false-negatives in *Shewanella *(i.e. proteins found by proteomics but not by signalp) may be a result of either distant phylogeny or the more liberal filters used to identify signal peptides.

Finally, we leverage comparative genomics and apply our data to 21 neighboring genomes, where we correct over 140 gene models. Instead of heuristics or a democratic voting scheme, we rely on experimentally validated protein sequences allowing us to confidently annotate orthologs across the genus. The heterogeneity of protein lengths in these orthologs highlights some of the difficulties surrounding comparative genomics and pangenomics. Although the sequences of *Y. pestis *are 90-95% identical, computational artifacts of the annotation process lead to several large differences between the protein calls. Furthermore, we saw diversity in the annotation of proteins split into multiple ORFs as mentioned above. All of these lead to inconsistency which can hamper a comparative genomics pipeline.

## Methods

### Mass Spectrometry Data

Spectra used in this report have been previously published. Briefly, the Pathogen Functional Genomics Resource Center (PFGRC) at JCVI generated ~15 million spectra from *Y. pestis KIM 6+*. Cell lysates were fractionated into cytoplasm, membrane and periplasmic components, and then run on 2 D gels, with spectrum acquisition on a Thermo LTQ [14,22,23]. All spectral data has been uploaded to NCBI's Peptidome http://www.ncbi.nlm.nih.gov/peptidome/.

### Data Processing

Our pipeline can be divided into distinct steps, with the overall goal of mapping peptides onto the genome and then inferring genome annotation (Figure 1). The first step is to prepare protein sequence databases by translating the RefSeq DNA into all six frames: genome NC_004088, and plasmids NC_004836, NC_004837, NC_004838. We do not make any restriction of open reading frames (ORFs). For example, we do not require a minimum length or the presence of a start codon. In addition to these *Yersinia *sequences, we append common contaminants (trypsin and keratin) to the database. To finish database preparation, we create a decoy database by shuffling each sequence [24].

We use the Inspect software to match spectra to peptide sequences from the protein database [25]. Search parameters: 25 tags/spectrum, cysteine + 57 fixed modification, and 3.0 Da parent mass tolerance. Following Inspect, we rescore peptide/spectrum matches (PSMs) with PepNovo [26]. We assign a p-value to PSMs based on the score distribution of hits to the target and decoy databases and set a 5% local false-discovery rate cutoff [21]. PSMs passing the cutoff are mapped back to their genomic ORF, and subjected to protein-level filters. To leverage the gel-based nature of our experimental set up, we extend the common 2 peptide heuristic to require 2 peptides per protein per gel spot. Manual inspection of the remaining PSMs (now grouped to their ORFs and reported as proteins) revealed a category of questionable results. We noticed PSMs mapping to low sequence-complexity regions, with significant overrepresentation of small amino acids (e.g. glycine or alanine). We believe that such sequences are likely to match noise spectra because the small mass allows for spurious peak matching. We also noticed that none of the PSMs in these objectionable clusters are tryptic. As trypsin was used to generate peptides for the experiment, we reject ORFs that lack any tryptic PSMs.

### Proteogenomic Improvement

We use PSMs passing the above filters to improve the RefSeq annotation of NC_004088 in three ways: adding novel gene models, correcting the start site of current models, and deleting dubious protein predictions. The updated KIM genome can be accessed seamlessly through any NCBI tool (e.g. blast) or downloaded directly ftp://ftp.ncbi.nih.gov/genomes/Bacteria/Yersinia_pestis_KIM_10_uid288/. We create a new gene model (novel gene) when at least 2 PSMs uniquely map to an ORF that lacks any protein annotation. We examine start sites when PSMs map upstream of and in frame with a current protein prediction. Through manual inspection, we adopted a simple heuristic, that the peptide supporting an extension must contain at least two amino acids upstream of the currently annotated start. With only a single amino acid upstream, it was possible for the amino acid to have the same mass as common modifications (e.g. glycine and carboxyamidomethyl (*CAM*) modification have a mass of 57).

We found many peptides which mapped to genomic loci containing multiple protein predictions. A closer inspection revealed that these loci often contained proteins with significant overlap. We marked genomic regions containing two or more genes with a 50 bp overlap as potentially *in conflict*. In our initial analysis, we divided conflicted loci into three categories defined by the relationship of the overlapping genes: convergent, divergent, unidirectional [27]. This delineation is attractive, because divergent and unidirectional overlaps can potentially be resolved by shortening one or both genes to remove the overlap. However, the extreme length and multiplicity of overlaps (some proteins overlapped two or three others) rendered this classification system awkward. Through visual analysis, we categorized conflicting loci according to the extent of overlap: 100% overlap, > 80% overlap, > 50% overlap, and < 50% overlap. It then became clear that in most instances, one of the overlapping genes was dubious and should simply be deleted.

When both genes had proteomic evidence, we asserted that both genes are real. In these instances, one or both of the genes was always overpredicted, i.e. was longer than homologs from other genomes. After correcting for this, the genes no longer overlapped in their coding regions. When only one gene had proteomic evidence, we tested whether the unobserved protein was a legitimate gene. Equivocal genes were determined using the following criteria. First, their extended sequence overlap (to a gene with proteomic support) is atypical, especially considering that many of them are completely contained within another protein's coding region. Second, they were sporadically predicted in bacterial genomes with poor and uncharacteristic conservation, while the proteomically supported gene had more compelling sequence conservation, was more consistently annotated within the genus/family, and had a generally broader phylogenetic distribution. Third, most of the dubious genes were short (median 95, mean 108). We note that 100 amino acids is the minimum for many genome annotation projects. We finally note that, if present, the computational evidence for asserting the equivocal gene in the original genome submission was weak (e.g. low sequence identity over short regions to phylogenetically distant organisms). Therefore, we term the equivocal gene as *dubious*, and have deleted it from the annotation.

### Mature Protein Annotation

To report a protein as containing a signal peptide, we started with proteins where the first observed peptide was not tryptic on its n-terminus, and was within 15-50 amino acids of the predicted start site [16]. Trypsin cuts after Arg and Lys; if the residue before the peptide is not Arg or Lys, then the peptide has a non-tryptic n-terminus. The region between the initial methionine and the first observed peptide is thus the putative signal peptide. We filtered this set using previously recognized signal peptide characteristics [17]. We required a hydrophobic patch of at least 8 amino acids, using the standard Kyte-Doolittle hydropathy index. We then examined the signal peptide terminus for the expected AxB cleavage motif (where A = [Ile, Val, Leu, Ala, Gly, Ser], B = [Ala, Gly, Ser]). Signal peptides matching these two criteria were considered *validated *by proteomics.

We ran signalp v3.0 on all proteins in the genome [28]. Proteins listed as containing a computationally predicted signal peptide are those with a D-score above 0.43 as recommended. When comparing signalp against proteomic evidence for signal peptide presence, we simply look gene by gene, whether proteomics or signalp or both list the protein as containing a signal peptide (and at the site of cleavage). Proteins with proteomic validation which have a signalp score below 0.43 were viewed as computational false-negatives. Conversely where peptides localize 5' of the signalp predicted cleave site (at least 4 residues upstream), we viewed these as computational false-positives.

### Proteogenomic Improvements in Other Genomes

We created orthology clusters of all genes in the 12 complete *Yersinia *genomes with the Sybil program [29]. Peptides observed in the KIM dataset were mapped onto the appropriate columns of an aligned cluster, and genome annotation improvements were inferred from this. For example, if a gene model in genome A was shorter than the orthologous member of the KIM genome, and peptide evidence from KIM mapped to the uncalled region, we checked for the ability to move the start site upstream in genome A. When genome improvements were possible (e.g. not precluded by stop codons) they were made and submitted to RefSeq. The same process was performed for the 9 Genbank annotations owned by JCVI. A list of all altered genes is presented in Additional File 3, Table S2. We are actively pursuing the ability to edit other genome annotations in collaboration with the genome owners.

## Authors' contributions

SHP designed the study, participated in the analysis and drafted the manuscript. SH participated in the analysis and generated the mass spectrometry data. RP participated in the analysis and coordination. All authors read and approved the final manuscript.

## Supplementary Material

Additional file 1 **Supplementary Figures**. This file contains supplementary figures S1 and S2. S1 is an image of a misannotated pseudogene. S2 is an image of prfB, which utilizes a programmed ribosomal frame shift. Click here for file

Additional file 2 **Supplementart Table S1, Signal Peptide Proteins**. This file contains information for all the proteins reported here as containing a signal peptide as observed by proteomics. Click here for file

Additional file 3 **Supplemental Table S2, All Protein Changes**. This file contains a list of all gene models altered, and what was altered. Click here for file

## References

[B1] Brubaker RR Sussman M Yersinia pestis Molecular Medical Microbiology 2002 3 London, UK: Academic Press 2033 2058 full_text

[B2] Aivaliotis M Gevaert K Falb M Tebbe A Konstantinidis K Bisle B Klein C Martens L Staes A Timmerman E Van Damme J Siedler F Pfeiffer F Vandekerckhove J Oesterhelt D Large-scale identification of N-terminal peptides in the halophilic archaea Halobacterium salinarum and Natronomonas pharaonis J Proteome Res 2007 6 2195 2204 10.1021/pr0700347 17444671

[B3] Gallien S Perrodou E Carapito C Deshayes C Reyrat JM Van Dorsselaer A Poch O Schaeffer C Lecompte O Ortho-proteogenomics: Multiple proteomes investigation through orthology and a new MS-based protocol Genome Res 2009 19 128 135 10.1101/gr.081901.108 18955433 PMC2612966

[B4] Gaasterland T Oprea M Whole-genome analysis: annotations and updates Curr Opin Struct Biol 2001 11 377 381 10.1016/S0959-440X(00)00213-X 11406390

[B5] Ouzounis CA Karp PD The past, present and future of genome-wide re-annotation Genome Biol 2002 3 COMMENT2001 10.1186/gb-2002-3-2-comment2001 11864365 PMC139008

[B6] Mann M Pandey A Use of mass spectrometry-derived data to annotate nucleotide and protein sequence databases Trends Biochem Sci 2001 26 54 61 10.1016/S0968-0004(00)01726-6 11165518

[B7] Ansong C Purvine SO Adkins JN Lipton MS Smith RD Proteogenomics: needs and roles to be filled by proteomics in genome annotation Brief Funct Genomic Proteomic 2008 7 50 62 10.1093/bfgp/eln010 18334489

[B8] Link AJ Robison K Church GM Comparing the predicted and observed properties of proteins encoded in the genome of Escherichia coli K-12 Electrophoresis 1997 18 1259 313 10.1002/elps.1150180807 9298646

[B9] Dandekar T Huynen M Regula JT Ueberle B Zimmermann CU Andrade MA Doerks T Sánchez-Pulido L Snel B Suyama M Yuan YP Herrmann R Bork P Re-annotating the Mycoplasma pneumoniae genome sequence: adding value, function and reading frames Nucleic Acids Res 2000 28 3278 3288 10.1093/nar/28.17.3278 10954595 PMC110705

[B10] Jungblut PR Müller EC Mattow J Kaufmann SH Proteomics reveals open reading frames in Mycobacterium tuberculosis H37Rv not predicted by genomics Infect Immun 2001 69 5905 5907 10.1128/IAI.69.9.5905-5907.2001 11500470 PMC98710

[B11] Jaffe JD Stange-Thomann N Smith C DeCaprio D Fisher S Butler J Calvo S Elkins T FitzGerald MG Hafez N Kodira CD Major J Wang S Wilkinson J Nicol R Nusbaum C Birren B Berg HC Church GM The complete genome and proteome of Mycoplasma mobile Genome Res 2004 14 1447 1461 10.1101/gr.2674004 15289470 PMC509254

[B12] Wei C Peng J Xiong Z Yang J Wang J Jin Q Subproteomic tools to increase genome annotation complexity Proteomics 2008 8 4209 4213 10.1002/pmic.200800226 18814329

[B13] Chain PS Hu P Malfatti SA Radnedge L Larimer F Vergez LM Worsham P Chu MC Andersen GL Complete genome sequence of Yersinia pestis strains Antiqua and Nepal516: evidence of gene reduction in an emerging pathogen J Bacteriol 2006 188 4453 4463 10.1128/JB.00124-06 16740952 PMC1482938

[B14] Pieper R Huang ST Clark DJ Robinson JM Parmar PP Alami H Bunai CL Perry RD Fleischmann RD Peterson SN Characterizing the dynamic nature of the Yersinia pestis periplasmic proteome in response to nutrient exhaustion and temperature change Proteomics 2008 8 1442 1458 10.1002/pmic.200700923 18383009

[B15] Sacerdot C Fayat G Dessen P Springer M Plumbridge JA Grunberg-Manago M Blanquet S Sequence of a 1.26-kb DNA fragment containing the structural gene for E.coli initiation factor IF3: presence of an AUU initiator codon EMBO J 1982 1 311 315 6325158 10.1002/j.1460-2075.1982.tb01166.x PMC553041

[B16] Gupta N Tanner S Jaitly N Adkins JN Lipton M Edwards R Romine M Osterman A Bafna V Smith RD Pevzner PA Whole proteome analysis of post-translational modifications: applications of mass-spectrometry for proteogenomic annotation Genome Res 2007 17 1362 1377 10.1101/gr.6427907 17690205 PMC1950905

[B17] Perlman D Halvorson HO A putative signal peptide recognition site and sequence in eukaryotic and prokaryotic signal peptides J Mol Biol 1983 167 391 409 10.1016/S0022-2836(83)80341-6 6345794

[B18] Deng W Burland V Plunkett G Boutin A Mayhew GF Liss P Perna NT Rose DJ Mau B Zhou S Schwartz DC Fetherston JD Lindler LE Brubaker RR Plano GV Straley SC McDonough KA Nilles ML Matson JS Blattner FR Perry RD Genome sequence of Yersinia pestis KIM J Bacteriol 2002 184 4601 4611 10.1128/JB.184.16.4601-4611.2002 12142430 PMC135232

[B19] Salzberg SL Genome re-annotation: a wiki solution? Genome Biol 2007 8 102 10.1186/gb-2007-8-6-r102 17274839 PMC1839116

[B20] Baudet M Ortet P Gaillard JC Fernandez B Guérin P Enjalbal C Subra G de Groot A Barakat M Dedieu A Armengaud J Proteomic-based refinement of Deinococcus deserti genome annotation reveals an unwonted use of non-canonical translation initiation codons Mol Cell Proteomics 2009 in press 19875382 10.1074/mcp.M900359-MCP200 PMC2830850

[B21] Castellana NE Payne SH Shen Z Stanke M Bafna V Briggs SP Discovery and revision of Arabidopsis genes by proteogenomics Proc Natl Acad Sci USA 2008 105 21034 21038 10.1073/pnas.0811066106 19098097 PMC2605632

[B22] Pieper R Huang ST Robinson JM Clark DJ Alami H Parmar PP Perry RD Fleischmann RD Peterson SN Temperature and growth phase influence the outer-membrane proteome and the expression of a type VI secretion system in Yersinia pestis Microbiology 2009 155 498 512 10.1099/mic.0.022160-0 19202098

[B23] Pieper R Huang ST Clark DJ Robinson JM Alami H Parmar PP Suh MJ Kuntumalla S Bunai CL Perry RD Fleischmann RD Peterson SN Integral and peripheral association of proteins and protein complexes with Yersinia pestis inner and outer membranes Proteome Sci 2009 7 5 10.1186/1477-5956-7-5 19228400 PMC2663777

[B24] Elias JE Gygi SP Target-decoy search strategy for increased confidence in large-scale protein identifications by mass spectrometry Nat Methods 2007 4 207 214 10.1038/nmeth1019 17327847

[B25] Tanner S Shu H Frank A Wang LC Zandi E Mumby M Pevzner PA Bafna V InsPecT: identification of posttranslationally modified peptides from tandem mass spectra Anal Chem 2005 77 4626 4639 10.1021/ac050102d 16013882

[B26] Frank AM A ranking-based scoring function for peptide-spectrum matches J Proteome Res 2009 8 2241 2252 10.1021/pr800678b 19231891 PMC2692183

[B27] Rogozin IB Spiridonov AN Sorokin AV Wolf YI Jordan IK Tatusov RL Koonin EV Purifying and directional selection in overlapping prokaryotic genes Trends Genet 2002 18 228 232 10.1016/S0168-9525(02)02649-5 12047938

[B28] Bendtsen JD Nielsen H von Heijne G Brunak S Improved prediction of signal peptides: SignalP 3.0 J Mol Biol 2004 340 783 795 10.1016/j.jmb.2004.05.028 15223320

[B29] Crabtree J Angiuoli SV Wortman JR White OR Sybil: methods and software for multiple genome comparison and visualization Methods Mol Biol 2007 408 93 108 full_text 18314579 10.1007/978-1-59745-547-3_6

