# Supplementary material for: A proteogenomic update to Yersinia: enhancing genome annotation
Source: BMC Genomics. 2010 Aug 5;11:460. doi: 10.1186/1471-2164-11-460 (PMC3091656; doi:10.1186/1471-2164-11-460)
Supplement: Additional file 1 — Supplementary Figures. This file contains supplementary figures S1 and S2. S1 is an image of a misannotated pseudogene. S2 is an image of prfB, which utilizes a programmed ribosomal frame shift. [file 1471-2164-11-460-S1.DOC]

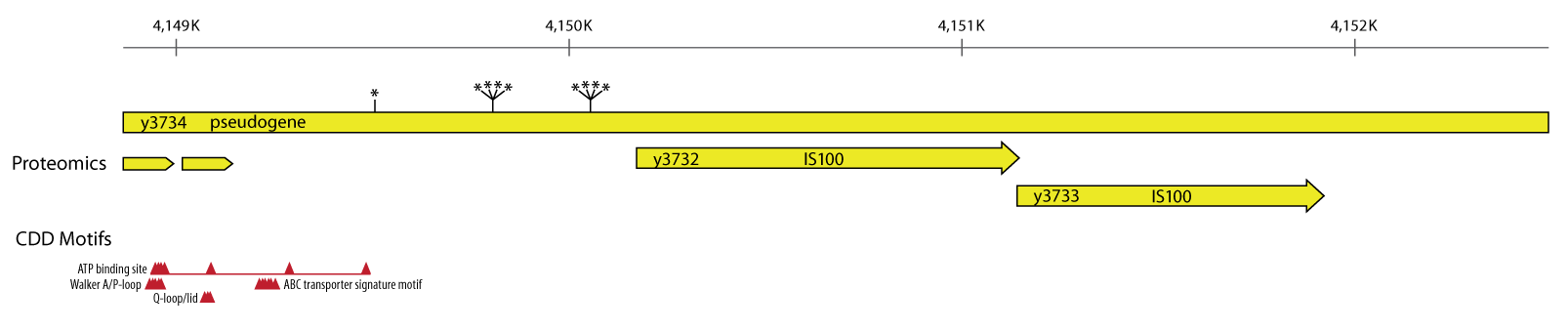


## Figure S1 - Misannotated Pseudogene

*Y. pestis KIM* gene y3734 is annotated as a pseudogene. There are both transposon insertions (IS elements y3733 and y3732) and small indel insertions which disrupt the protein sequence. Stop codons in the original frame are marked with stars. Peptides from proteomics are shown near the n-terminus. Sequence motifs present in the observed protein are shown. Other motifs required for proper function were not found inframe, and are not shown.


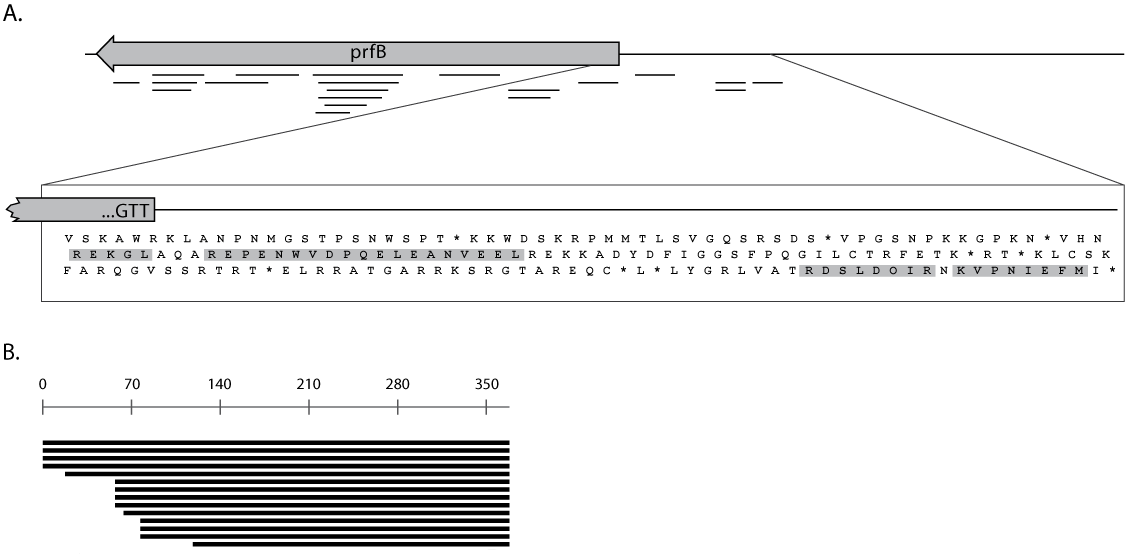


## Supplemental Figure 2 - Ribosomal Frame Shift

(A) Peptide chain release factor II, prfB, is shown with peptides mapping to the region. Zoom-in shows peptides mapping to both the bottom and middle frame. (B) The corrected pfrB sequence is aligned against all other non-redundant prfB Yersinia sequences. Most sequences within the genus are mispredicted by > 50 amino acids. Similarly disparate annotations can be seen in numerous genera in the bacterial tree of life (e.g. Salmonella, Bacillus).
